# Supplementary material for: Risk of hospitalization and mortality due to COVID-19 in people with obesity: An analysis of data from a Brazilian state
Source: PLoS One. 2022 Mar 4;17(3):e0263723. doi: 10.1371/journal.pone.0263723 (PMC8896734; doi:10.1371/journal.pone.0263723)
Supplement: Attachment — Submitted filename: Letter to the editor.docx [file pone.0263723.s001.docx]

Dear Editor

We greatly appreciate the reading and suggestions made in our manuscript “Risk of hospitalization and mortality due to COVID-19 in people with obesity: An analysis of data from a Brazilian state”. The manuscript was modified according to the reviewers' suggestions and below we answer the questions presented point by point.

**Reviewer #1:**

General:
Please adhere to the STROBE guidelines for reporting of observational studies: <https://www.equator-network.org/reporting-guidelines/strobe/>

The article was reviewed based on the STROBE guidelines. When absent, the themes presented in the checklist were included.

Introduction

1) Please explain why your study is needed. It seems that the question your study tries to answer is already answered.

This study is necessary due to the scarcity of studies that discuss the relationship between obesity and COVID-19 in Brazil. Considering that the pandemic is still out of control in the country, it is important to assess the risk for this population. These data were included in the article.

2) Regarding the objective: “… this study aims to assess the risk of hospitalization and mortality due to COVID-19 in people with obesity based on data from Espírito Santo residents, Brazil.” The study methods and results imply a more wide approach. Please adjust the aims to be consistent with the rest of the manuscript.

The aims were adjusted and rewritten.

Methods

3) How are patients includes in this database, what were the selection criteria? How many were eligible and how many were not included?

All suspected cases (under clinical and/or laboratory investigation) or confirmed cases of COVID-19 in the state of Espírito Santo are registered in this database. Registration is carried out through the notification form filled in at all health services in the state. For this research, only confirmed cases were selected.

4) Describe methods of follow-up? How long was the follow-up?

People are monitored by the Health Unit that made the registration. Since the beginning of the pandemic (03/05/2021, when the state registered the first case) until September 10, 2020, 118,138 confirmed cases were registered by COVID-19 in the state of Espírito Santo. People who still had no evolution (cure or death) described in the database were excluded, and thus data from 59,698 people were analyzed, and of these, 3025 people were obese. As this study is cross-sectional, there was no follow-up of people registered with COVID-19. Data were analyzed from the state health department registry. Data were accessed and downloaded on 10/09/2021 and analyzed according to the criteria described.

5) How was race/ ethnicity determined? Self reported? Determined by physician? What do you mean by yellow ect?

Race/ethnicity was obtained by self-report or determined by the health professional when the individual was unable to account for the severity of the disease. Yellow people are people from Asia who have specific characteristics and ways of life. When performing the review of the results, the yellow and indigenous people were excluded from the analysis because they are a population with specific characteristics.

6) Regarding death, there maybe patients remaining in hospital that have no yet reached an outcome (either death or discharged). This means there is a high risk of (non)-differential misclassification which could lead to bias.

No. We selected for this study people confirmed for COVID-19 who had the evolution of the disease closed as cure or death. People who were still undergoing treatment were excluded.

7) Are the authors using a causal or a prognostic approach? Presently, the “adjusted model” is not suitable for causal interpretation nor is it adequately validated for use as a prognostic model. Please see also these references below:
doi: 10.1097/EDE.0000000000001258

DOI: 10.1097/EDE.0000000000001259

8) A such, there also seems to be a Table 2 fallacy problem with the analyses:
DOI: 10.1093/aje/kws412

The symptoms of runny nose, sore throat, diarrhea and headache showed counterintuitive associations, possibly due to confounding variables not properly controlled, resulting in statistically significant associations, giving the false idea of a "protective effect" for both hospitalization and death due to COVID-19.

Results

9) Please report, according to STROBE item 13: “numbers of individuals at each stage of study—eg numbers potentially eligible, examined for eligibility, confirmed eligible, included in the study, completing follow-up, and analysed” A flow diagram is highly recommended.

A flow diagram was prepared based on STROBE and the number of individuals in each stage of the study was described.

10) How many patients had missing data and how was this handled?

Data from 5,669 patients were missing, as described in the flow diagram that describes each step of the selection of individuals. These records were excluded from the analysis.

11) Why present OR when you can present RR?

We are presenting the OR instead of the RR, as the study design is cross-sectional, that is, the study population was observed only once and information regarding the outcome and exposure was collected at the same time. RR is usually used in clinical trials or in cohorts.

12) Table 2-5: It would be helpful to add the number of patients to each table, e.g. n of x men were hospitalized and n of y women were hospitalized.

The number of patients was included.

Discussion

13) The discussion could benefit from a thorough limitations section.

Limitations of the study were added.

**Reviewer #2:**

Introduction
*I would make the introduction much shorter. In specific, I would remove the first three paragraphs since the general information provided there is well-known. I would start directly with risk factors/obesity.

The three paragraphs have been removed.

Methods
*This section needs English language editing.*Authors need to make sure they report all component of STROBE checklist. *Inclusion and exclusion criteria can be defined more clearly.*How was obesity defined? Did researchers rely on documentation or they used BMI or other tool?

In this second version the English has been revised. The STROBE checklist items were observed. The inclusion and exclusion criteria were presented more clearly and included in the flow diagram. Regarding BMI, data refer to weight and height self-reported or registered by the health professional, according to the structure of the health service.

Results
*This section needs English language editing, as well. *Would change obese patients to patients with obesity

The text has been changed to "people with obesity".

*Table 1: What is yellow color/race?

Yellow people are people from Asia who have specific characteristics and ways of life. When performing the review of the results, the yellow and indigenous people were excluded from the analysis because they are a population with specific characteristics. The study data were analyzed again with modifications in the method (such as the exclusion of yellow and indigenous people) and thus the entire results section was rewritten. This did not change the study's conclusions, only the values.

*Do the rest of the tables present univariate or multivariate associations? This should be clear in the text and the table notes. *Why some variables were placed in the regression, while others were not?

The tables show univariate association. This was described in the method.

**King Regards,**

**Dr. Erika Cardoso dos Reis**
