# Supplementary material for: Risk of hospitalization and mortality due to COVID-19 in people with obesity: An analysis of data from a Brazilian state
Source: PLoS One. 2022 Mar 4;17(3):e0263723. doi: 10.1371/journal.pone.0263723 (PMC8896734; doi:10.1371/journal.pone.0263723)
Supplement: Attachment — Submitted filename: Response to Reviewers Jan-22.docx [file pone.0263723.s003.docx]

Dear Editor

We greatly appreciate the new reading and suggestions made in our manuscript “Risk of hospitalization and mortality due to COVID-19 in people with obesity: An analysis of data from a Brazilian state”. The manuscript was modified according to the reviewers' suggestions and below we answer the questions presented point by point.

Below, we respond point by point to the comments and issues mentioned by the reviewers.

**Reviewers' comments:**

Please make the following changes to use more inclusive terminology:
Yellow is still used to describe Asian populations in the flow chart. Yellow is not a race or ethnicity, please change.

R: The term Yellow was changed to Asian populations.

Please replace "illiterate" used throughout the manuscript with "No education"

R: The term illiterate was changed to No education.

**Comment 1:**

Regarding Previous comment and response 6 (see below), the people who were still under treatment and who were excluded could have a different risk of dying than the included people and they can have a different distribution of co-variates. Hence there is a high risk of differential misclassification especially considering the large proportion of patients that were excluded (49%): 59.698 of 118.138 people were included.

Previous comment and response 6:

Regarding death, there may be patients remaining in hospital that have no yet reached an outcome (either death or discharged). This means there is a high risk of (non)- differential misclassification which could lead to bias. No. We selected for this study people confirmed for COVID-19 who had the evolution of the disease closed as cure or death. People who were still undergoing treatment were
excluded.

R: This is a cross-sectional study and as it is not intended to be a longitudinal study. For this reason, it cannot follow the evolution of the disease in individuals. Considering that individuals not included in this analysis (because their case was not closed) could have a higher risk of mortality, we would have to consider that these individuals could also have a lower risk of mortality and not go to the ICU. Because this is also a possibility and it cannot be considered a bias in the cross-sectional study.

**Comment 2:**

Previous comment 7 (see below) has not been addressed by the authors.

Previous comment 7:

Are the authors using a causal or a prognostic approach? Presently, the “adjusted model” is not suitable for causal interpretation nor is it adequately validated for use as a prognostic model. Please see also these references below:

doi: 10.1097/EDE.0000000000001258.

DOI: 10.1097/EDE.0000000000001259.

R: Only association without causality and prognosis was measured.

**Comment 3:**

Regarding previous comment and response 8, the potential Table 2 fallacy problem has not been addressed by the authors (see below).

Previous comment and response 8:

A such, there also seems to be a Table 2 fallacy problem with the analyses: DOI: 10.1093/aje/kws412 The symptoms of runny nose, sore throat, diarrhea and headache showed counterintuitive associations, possibly due to confounding variables not properly controlled, resulting in statistically significant associations, giving the false idea of a "protective effect" for both hospitalization and death due to COVID-19.

R: These are symptoms of mild COVID-19 and that is why in the study it has a protective effect. Because people who have these symptoms on hospitalization may have a lower chance of death.

**King Regards,**

**Dr. Erika Cardoso dos Reis**
